# Mechanochemical synthesis of metal oxide nanoparticles

**DOI:** 10.1038/s42004-021-00582-3

**Published:** 2021-10-12

**Authors:** Takuya Tsuzuki

**Affiliations:** School of Engineering, Australian National University, Canberra, ACT 2601 Australia

**Keywords:** Design, synthesis and processing, Nanoparticle synthesis

## Abstract

In the last decades, mechanochemical processing has emerged as a sustainable method for the large-scale production of a variety of nanomaterials. In particular, mechanochemical synthesis can afford well-dispersed metal-oxide nanoparticles, which are used in wide-ranging applications including energy storage and conversion, environmental monitoring, or biomedical uses. This article reviews recent progress in the mechanochemical synthesis of metal-oxide nanoparticles, explores reaction mechanisms, and contrasts the influence of chosen process parameters on the properties of end products. The role of choice of reaction pathway, as well as advantages and limitations compared to other synthesis methods are discussed. A prospect for future development of this synthetic method is proposed.

## Abstract (web summary)

Mechanochemistry is driven by mechanical energy input, which often alleviates the need for solvents or heating to achieve desired products. Here, the author reviews mechanochemical syntheses of metal-oxide nanoparticles, exploring reaction mechanisms, and contrasting the influence of process parameters on the properties of end products.

## Introduction

Nanoparticles are a new class of engineering materials. They are small particulate materials with a characteristic length scale of < 100 nm. Nanoparticles exhibit new or enhanced size-dependent properties compared with the larger counterparts of the same material. The unique properties are governed by the size and shape of the nanoparticles. Thus, the control of size and shape enables the design of nanoparticles with specific properties desired in their applications. Owing to these unique properties of nanoparticles and recent development in their synthesis methods, the current and potential applications of nanoparticles are rapidly growing. Among many types of nanoparticles, metal-oxide nanoparticles are widely used in an extremely broad range of industries, including the manufacture of integrated circuits, biomedical and cancer treatment, renewable energy, environmental protection, pharmaceuticals, personal care, surface coatings, plastics, textiles, food, building materials, electronics and automotives^1^.

A small variety of metal-oxide nanoparticles such as fumed silica and TiO_2_ pigments have been produced as early as the mid-twentieth century. However, it is in the last three decades that significant research efforts have been made to develop production methods for metal-oxide nanoparticles. As a result, a considerable number of synthesis techniques are currently used in laboratories worldwide. Among them, mechanochemical processing has emerged as a unique technique to produce metal-oxide nanoparticles. Due to the simplicity in the operation parameters, the adaptation of mechanochemical synthesis from lab-benchtop synthesis to commercial-scale production took < 8 years. In the last few years, the awareness towards the potential environmental benefits of mechanochemistry has increased; mechanochemical processing allows chemical reactions that normally occur at elevated temperatures, to be induced at near-room temperature without the use of organic solvent. This article reviews the recent progress in the mechanochemical processing of metal-oxide nanoparticles. It discusses the uniqueness of mechanochemical synthesis techniques, the importance of selecting appropriate reaction pathways, and the roles of process parameters that govern the properties of resulting nanoparticles. This article focuses on the production of well-separated metal-oxide nanoparticles via mechanochemical activation of solid-state displacement reactions. The synthesis of nano-grained, nanostructured or highly aggregated metal oxides by mechanochemical means are outside the scope of this article^2,3^. We first discuss the mechanochemical synthesis of nanomaterials and then review the applications of mechanochemistry to the production of a variety of metal-oxide nanoparticles.

## Mechanochemical synthesis

### Mechanochemical processing

Mechanochemistry is concerned with the chemical reactions and structural changes induced by mechanical energy^4^. Mechanochemistry has been utilised by humans for millenniums^5,6^. In the modern world, many industries embrace this technology, including mining, building, pharmaceutical and manufacturing sectors^6^. The first systematic investigation of mechanochemistry was conducted in the nineteenth century^7^, and mechanochemistry was recognised as a branch of chemistry by Wilhelm Ostwald around the turn of the twentieth century. Since then, the discipline has been made steady progress. However, it is due to its aspects of green chemistry that mechanochemistry has seen a recent resurgence^8–11^. In 2019, the International Union of Pure and Applied Chemistry (IUPAC) named mechanochemistry as one of the top ‘10 chemical innovations that will change our world’, in honour of IUPAC’s 100th anniversary^12^.

The advancement of modern technology realised different types of high-energy ball mills with mechanical energy output (energy density) high enough to induce many chemical reactions. As such, in mechanochemistry, ball mills are often utilised as a chemical reactor. Because of this reason, mechanochemical processing is also called ‘reactive milling’^13^. Attrition mills, planetary mills and shaker mills are common types of mills used in mechanochemistry. The former two types of mills are also used for the commercial large-scale production of metal-oxide nanoparticles.

The mechanisms to mechanically activate chemical reactions have been the subject of many studies. Mechanical energy input repeatedly causes the shifts of atoms from the equilibrium stable positions and in turn the changes of bond lengths and angles, and, in some cases, the excitation of electron subsystems^14^. This leads to the creation of defects, amorphization or metastable phases to accumulate energy that is released to rapture chemical bonds and causes chemical reactions^5^. However, the detailed study on the mechanochemical activation during ball milling is not straightforward, because of the short duration of each collision event and the localised character of the event in terms of heat and pressure^15^. Nevertheless, in situ measurements have been attempted^16–18^, and thermodynamical analyses^19^ and kinetic modelling^20^ have been conducted to understand the reaction processes. Detailed discussions on mechanochemistry are reviewed elsewhere^15,19,21–31^.

A wide variety of materials have been produced using mechanochemical processing^29^. They include metals and their alloys, oxides and other chalcogenides, refractory materials such as carbides and silicides, common salts such as carbonates and phosphates^32^. The ability of mechanochemical processing is not limited to the synthesis of inorganic materials. Metal organic frameworks^33^, organometallic complexes^34,35^ and organic compounds^29,36^ can also be produced using mechanochemistry. Moreover, the production of complex structures^37^ such as hybrid materials consisting of a core of inorganic materials and a shell of organic materials^38^ was demonstrated by mechanochemical processing. The usefulness of mechanochemical processing to produce nanostructured materials was also recognised at an early development stage of nanotechnology^39^.

### Application of mechanochemical processing to the synthesis of nanoparticles

The methods to synthesise nanoparticles are commonly classified into two approaches; top-down and bottom-up^1^. Top-down approaches are an extension of the traditional methods used to produce ultrafine powders from bulk raw materials by grinding them down to smaller pieces^40^, either in air, in a liquid media^41^ or in a solid diluent phase. In bottom-up approaches, nanoparticles are formed from small building blocks such as atoms or molecules under vacuum or in a gas, liquid or solid media. Mechanochemical processing is one of the bottom-up approaches to synthesise nanoparticles.

Mechanical pulverisation (top-down approach) is occasionally misunderstood as mechanochemical processing. For example, one of the ways to obtain metal-oxide nanoparticles is the pulverisation of precursor materials by ball milling in a solid matrix and subsequent heat treatment to decompose the precursors into metal oxides^42–47^. However, unless chemical changes are induced to the precursor particles during milling, the process is not mechanochemical. Likewise, mechanochemical processing is often misunderstood as a top-down approach, due to the grinding process involved^48^. The grinding process is involved in mechanochemical processing only to the pulverisation of raw reactant materials. The essence of mechanochemical processing is the induction of chemical reactions between raw materials by the input of mechanical energy^49,50^.

**Fig. 1:**
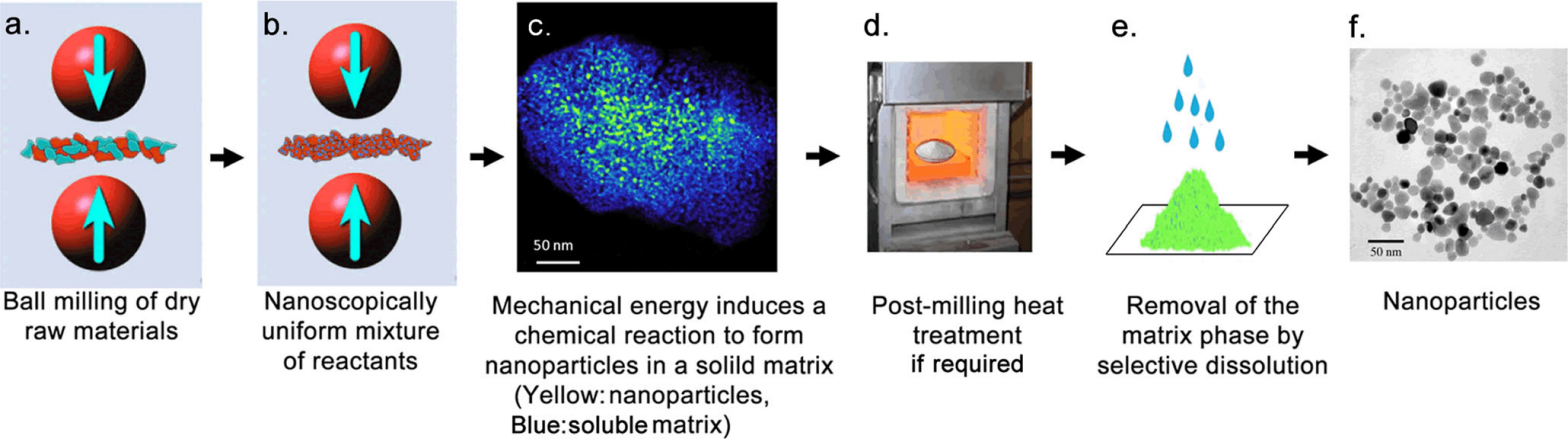
Typical steps of mechanochemical processing to produce nanoparticles via a solid-state displacement reaction. **a** Dry raw reactant materials are placed in a milling container along with milling balls. **b** During ball milling, repeated fracture and welding of the raw materials result in the formation of a nanocomposite of the reactants. **c** Mechanical energy input into the reactant nanocomposite induces a chemical reaction to produce nanoparticles in a solid matrix^48^ (reproduced with permission from Springer). **d** If necessary, post-milling heat treatment is conducted to complete the chemical reaction or to control the size, shape or crystallinity of nanoparticles. **e** Soluble by-product matrix phase is removed. **f** Final nanoparticle products.

During ball/powder collision events, repeated deformation, fracture and welding of reactant materials occur. This leads to the formation of a nanoscale composite structure of the reactant materials^48^. Within the nanocomposite structure, chemical reactions are initiated across the grain boundaries of the adjoining reactant phases. This condition drastically enhances the reaction kinetics mainly in the following three aspects. Firstly, nanoscale grain sizes significantly reduce the diffusion length of reactant atoms^51^. Secondly, the high residual energy in the form of structural defects or radicals in the reactant nanocomposite reduces the reaction threshold energy. Thirdly, repeated welding and fracture of raw materials continuously generates fresh reaction surfaces. Thus, mechanochemical processing enables the near-room-temperature induction of chemical reactions that otherwise occur only at elevated temperatures after prolonged heat treatment (Fig. 1). Even in the cases where the chemical reaction does not complete during milling, the reaction can be completed by post-milling heat treatment at only moderate temperatures, because the reaction threshold energy is substantially lowered during mechanical treatment. The theoretical analysis of mechanochemically induced reaction process was reported elsewhere^52–54^.

In dry mechanochemical synthesis of nanoparticles, solid-state displacement reactions are often utilised^55,56^. The reaction is represented by the following equation:

1 $${{{{{{{\mathrm{A}}}}}}}}^ + {{{{{{{\mathrm{B}}}}}}}}^ - + {{{{{{{\mathrm{C}}}}}}}}^ + {{{{{{{\mathrm{D}}}}}}}}^ - \to {{{{{{{\mathrm{A}}}}}}}}^ + {{{{{{{\mathrm{D}}}}}}}}^ - + {{{{{{{\mathrm{C}}}}}}}}^ + {{{{{{{\mathrm{B}}}}}}}}^ -$$

Often dry-powder forms of raw materials are used. Mechanochemical processing of the raw materials results in the solid-state precipitation of nanoparticles in a solid by-product matrix. Upon completion of the reaction, nanoparticles can be collected by selectively removing the by-product matrix phase.

### Advantages and limitations of the mechanochemical synthesis of nanoparticles

The aspects of green chemistry are regarded as the advantage of mechanochemical synthesis. Mechanochemical processing allows the production of metal-oxide nanoparticles without using organic solvents or high temperatures, thus has the potential in reducing environmental footprint^8,9^. In contrast, many liquid-phase synthesis techniques require organic solvents and vapour-phase synthesis uses organic precursors. Water-based liquid-phase synthesis tends to have a high reaction rate that causes difficulty in the precise control of particle-size distribution without the use of surfactants^1^. Nevertheless, high-energy ball milling may require high-energy consumption^57^. A detailed comparison in environmental benefits between different bottom-up approaches to obtain metal-oxide nanoparticles should be conducted using a rigorous life-cycle analysis^58^. Nevertheless, it was demonstrated that the mechano-synthesis of organometallic species displays a low E-factor and low-process mass intensity compared to conventional solution methods^59^.

**Fig. 2:**
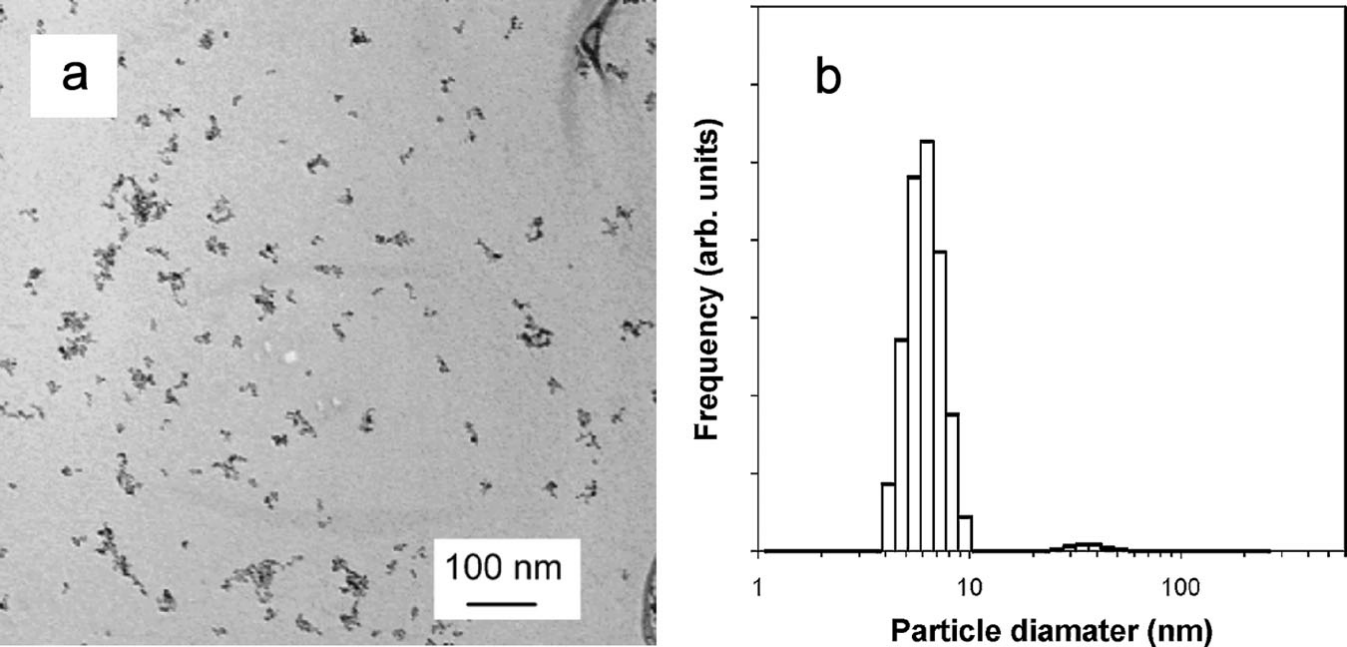
Mechanochemically synthesised γ-Fe_2_O_3_ single-crystalline nanoparticles^93^. **a** Transmission electron micrograph. **b** Particle-size distribution obtained from electron micrographs. Reproduced with permission from Elsevier.

Another advantage of mechanochemical techniques is that it allows the formation of nanoparticles with a low degree of agglomeration (Fig. 2). This is possible because a solid by-product matrix acts as a physical barrier between particles during the particle-growth stage. In contrast, the liquid- or vapour-phase synthesis of nanoparticles can lead to particle agglomeration because of the lack of a solid physical barrier between particles during the particle-growth stage. Liquid-phase synthesis methods can utilise surfactants or micelles to inhibit particle agglomeration, but the use of surfactants or micelles leads to limited options in the surface treatment of nanoparticles at the application stage. Without a solid barrier, liquid- or vapour-phase synthesis needs to resort to highly diluted conditions to produce nanoparticles to avoid particle agglomeration, which leads to a decreased product yield.

Mechanochemial synthesis has other advantages such as relatively simple operation, ease of scale-up, and ease to create a uniform reaction environment that leads to uniform size and shape of nanoparticles^55,56^. In contrast, when liquid-phase techniques are scaled-up, the reaction environment is often larger than the size of a beaker and its homogeneity becomes an issue.

The disadvantages of mechanochemical synthesis over other methods include the following:

- The end products are subject to contamination from the by-product phase, milling balls or milling containers. In contrast, vapour-phase synthesis can produce metal-oxide nanoparticles with extremely high purity^1^.
- The end product may suffer from low crystallinity, due to the residual defects and amorphization caused by mechanical energy input. In contrast, vapour-phase synthesis can produce metal-oxide nanoparticles with extremely high crystallinity due to the high temperature involved. Although the crystallinity of mechanochemically synthesised particles can be improved by post-milling annealing, the heating process may cause particle agglomeration or the migration of impurities from the by-product phase into nanoparticles^1^.
- The production of highly soluble metal oxides that decomposes upon contact with washing liquids, cannot be separated from the by-products easily. For example, B_2_O_3_ is soluble in water and many organic solvents, so that the removal of a by-product phase needs to rely on sublimation or other physical means instead of a washing process^60^.

## Influence of production parameters on the characteristics of end products

### Ball size

Ball size influences the mechanical energy input. The increase in ball size leads to increased input-energy density per collision event. Thus, generally speaking, larger balls can induce desired chemical reactions in a shorter milling time than smaller balls. The density of milling balls also influences the input-energy density per collision event. However, the choice of materials for milling balls are often limited. In high-energy ball mills, milling balls are often made of the same material as the milling chamber in order to reduce the contamination level arising from the wear of the chamber and balls^61^. The chambers of industrial high-energy ball mills are often constructed with steel and hence hardened steel balls or stainless steel balls are frequently used^61^. Ceramic milling balls with a high density, such as tungsten carbide, are commercially available but, due to their brittle nature, a significant amount of ball fragments could contaminate the end products after high-energy ball milling^62^. Michalchuk et al.^63^ demonstrated that, for the co-crystallisation of organic compounds, both ball size and ball mass play critical roles in determining the reaction rate. However, to date, there is no similar study reported for the production of metal-oxide nanoparticles.

In some cases, large milling balls input mechanical energy high enough to cause self-propagating exothermic reactions, the so-called combustion events, within the milling vessel^64,65^. The occurrence of combustion is not desirable for the production of nanoparticles, as the combustion causes a significant temperature increase that results in melting or vaporisation of milled powders or agglomeration of resulting particles^66^. Therefore, it is critical to suppress the occurrence of combustion for the production of uniform nanoparticles with little agglomeration. Reducing the ball size can delay or eliminate combustion by decreasing the mechanical energy density during ball-to-powder collision events^66,67^.

Ball size also affects the size of nanoparticles. Smaller balls lead to smaller nanoparticles. This is because (i) smaller milling balls lead to finer grinding of raw materials^68^ and (ii) smaller energy input causes shorter diffusion length of the reactant atoms. For example, although not a metal oxide, CdS quantum dots were synthesised via a mechanochemical reaction CdCl_2_ + Na_2_S → CdS + 2NaCl^55^. Milling was performed with a shaker mill for 1 h using a ball-to-powder mass ratio of 10:1 and steel balls of 4.8, 6.4, 9.5 and 12.6 mm in diameter. As the ball size was reduced from 12.6 to 4.8 mm, the average size of the resulting CdS quantum dots decreased from 8.2 to 4.3 nm. Since the particle sizes were below the exciton Bohr diameter of CdS, the bandgap energy of CdS increased through the quantum-confinement effect, as the ball size decreased.

### Addition of a diluent in the starting powder mixture

The addition of an inert diluent serves two critical purposes to obtain nanoparticles with a low degree of agglomeration. Firstly, it suppresses combustion events during milling^67^. As discussed earlier, combustion causes temperature increase that results in large primary and secondary particles. If the diluent phase is inert and does not react with reactants, the diluent phase separates reactants from each other, thus influences the reaction kinetics by decreasing the collision frequency between the reactants during milling and reducing the effective reaction areas. In addition, the diluent phase acts as a heat sink to moderate any increase in the local temperatures associated with the reaction enthalpy^67^. The diluent also absorbs the mechanical energy. As a result, the addition of a diluent is effective in preventing combustion events during milling^69,70^. For example, when dry powders of CeCl_3_ and NaOH were milled together to produce CeO_2_ via the reaction CeCl_3_ + 3NaOH → CeO_2_ + 3NaCl + 1.5H_2_O under a high-purity argon gas atmosphere, a combustion event occurred within 2 min of milling and large aggregates of CeO_2_ (~500 nm in diameter) were obtained. In contrast, the addition of 12 moles of NaCl diluent to make up the starting powder mixture of CeCl_3_ + 3NaOH + 12NaCl prevented combustion from occurring and resulted in CeO_2_ nanoparticles of ~10 nm in diameter with a low degree of agglomeration^70^. However, the suppression of reaction kinetics leads to a longer milling time to complete the reaction^51^.

The second purpose of the addition of an inert diluent is to decrease the volume fraction of nanoparticles in the product phase. When the volume fraction of particles in the product phase is larger than 20%, the particles are no longer separated from each other by the by-product matrix, resulting in the formation of large aggregates^71^. By contrast, when the volume fraction of nanoparticles is < 20%, the particles tend to have little agglomeration^56^.

The volume fraction of the nanoparticles in the product phase also influence the size of nanoparticles. A smaller volume fraction results in smaller particle sizes^55,71^. For example, during mechanochemical synthesis of SnO_2_ nanoparticles via the reaction SnCl_2_ + Ca(OH)_2_ + 0.5O_2_(g) → SnO_2_ + CaCl_2_ + H_2_O(g), 29% of SnO_2_ volume fraction led to the formation of 1-μm sized aggregates, whereas 9% of volume fraction resulted in separated nanoparticles of ~30 nm in diameter^71^. Since smaller reactant particles lead to smaller product particles^51^, the size reduction of nanoparticles by increasing the volume fraction of NaCl may be attributed to the reduction of reactant particles during milling. In addition, the degree of crystallite growth decreases with increased levels of diluent, because the heat-sink effect of the diluent phase lowers the local adiabatic reaction temperature, leading to shorter diffusion lengths of reactant ions^72^.

Recently, NaCl was shown to play an active role, instead of being an inert diluent, in the mechanochemical processing of metal-oxide nanostructures. Shu et al.^73^ suggested that metal-chloride stating reactants can mechanochemically form a solid solution with NaCl through the so-called iron-sharing ability of NaCl, which leads to the improvement of oxide microstructure and the preparation of porous materials. This implies that NaCl should not be blindly regarded as an inert diluent. The possibility of NaCl to form a solid solution with metal-halide precursors and its influence on the reaction pathways and the characteristics of resulting nanoparticles need to be elucidated in future studies.

## Mechanochemical synthesis of metal-oxide nanoparticles

### Binary metal oxides

For the successful mechanochemical synthesis of metal-oxide nanoparticles, it is critical to carefully select suitable chemical reactions. The choice of raw materials influences the solubility of the by-product phase and the particle-matrix volume ratio. As the source of metal ions in metal-oxide nanopowder, metal salts such as chlorides are frequently used because of their readily available nature. Alkali-earth oxides are considered as an adequate counter- reactant to metal chlorides because they transform into water-soluble alkali-earth chlorides upon reaction with metal chlorides, whereby enabling easy, safe and green removal of the by-product phase. However, alkali-earth oxides such as CaO are often found non-reactive during ball milling, even when the Gibbs free energy change of the reaction is largely negative.

For example, ball milling of ZrCl_4_ and CaO dry powders does not result in the induction of mechanochemical reaction ZrCl_4_ + 2CaO → ZrO_2_ + 2CaCl_2_ (dG = −440 kJ), thus requiring a post-milling heat treatment at 300 °C for 1 h to complete the reaction^74^. Subsequent removal of CaCl_2_ yielded c-ZrO_2_ nanoparticles of ~10 nm in diameter with a low degree of agglomeration. Interestingly, the use of MgO instead of CaO resulted in the induction of the reaction ZrCl_4_ + 2MgO → ZrO_2_ + 2MgCl_2_ (dG = −198 kJ) during ball milling^56^. The difference between CaO and MgO may be attributable to the milling characteristics of by-products; MgCl_2_ is more brittle and mills into fine powders easier than CaCl_2_ hence affecting the reaction kinetics.

Although careful handling is required due to their hygroscopic and caustic nature, alkali-metal oxides can also be used as the counter-reactants to metal salts. For example, García-Pacheco et al.^75^ produced CoO and Co_3_O_4_ nanoparticles using a solid-state dry reaction CoCO_3_ + Na_2_O → CoO + Na_2_CO_3_ (dG = −250 kJ) in a shaker mill under an inert-gas atmosphere. Co_3_O_4_ was obtained by the post-milling oxidation of CoO. The resulting nanoparticles were soft agglomerates with primary particle sizes between 15 and 20 nm. However, the ball milling of ZrCl_4_ and Li_2_O did not result in the induction of a chemical reaction ZrCl_4_ + 2Li_2_O → ZrO_2_ + 4LiCl, despite the large Gibbs free energy change (−561 kJ), thus a post-milling heat treatment was required to produce ZrO_2_ nanoparticles of ~40 nm in diameter^76^.

Another approach to synthesise metal-oxide nanoparticles is to produce a precursor of metal oxides in a form of nanoparticles via mechanochemical processing followed by the thermal decomposition of the precursor nanoparticles into metal oxides by heat treatment at a moderate temperature while the precursor particles are embedded in the solid by-product matrix to avoid particle agglomeration. Examples of potential precursors include metal hydroxides, carbonates, acetate, oxalate, nitrates and sulphates, though nitrates and sulphates are not preferred because their decomposition generates toxic gases.

This approach has two advantages over the use of alkali-earth oxide reactants. The first advantage is that the counter-reactants to form the precursor metal salts, for example, Ca(OH)_2_ to form a metal-hydroxide precursor, are generally more reactive than alkali-earth oxides, which increases the chance for the reaction to complete during ball milling without post heat treatment. The second advantage is that the transition of mechanochemically synthesised precursors into metal oxides accompanies volume reduction, allowing the resulting metal-oxide nanoparticles to become smaller than the precursor nanoparticles. However, the second advantage is normally ineffective, as the heat treatment itself causes particle growth^70^.

For example, the production of Fe_2_O_3_ nanoparticles by mechanochemical milling was attempted via the reaction 2FeCl_3_ + 3CaO → Fe_2_O_3_ + 3CaCl_2_ (dG = −508 kJ) in a shaker mill, but the reaction did not occur during milling for 24 h^77^. In contrast, the milling of FeCl_3_ and Ca(OH)_2_ for 24 h resulted in the formation of FeOOH in an amorphous CaCl_2_ matrix, despite the lower Gibbs free energy change (−334 kJ) of the reaction 2FeCl_3_ + 3Ca(OH)_2_ → FeOOH + 3CaCl_2_ + 2H_2_O^77^. This is attributable to the better milling ability of Ca(OH)_2_ than CaO and demonstrates the importance of kinetic factors on the occurrence of mechanochemical reactions. The FeOOH phase was subsequently converted to Fe_2_O_3_ upon heat treatment at 200 °C. The removal of CaCl_2_ resulted in Fe_2_O_3_ nanoparticles of ~100 nm in diameter. The use of NaOH was also effective in completing the chemical reaction during milling to obtain Fe_2_O_3_ from FeCl_3_^78^. Other oxide nanoparticles including CeO_2_ (10 nm)^70^, Fe_3_O_4_ (10 nm)^79,80^, and ZrO_2_ (~10 nm)^81,82^ were formed in a similar manner using hydroxide counter-reactants. However, the use of alkali-metal hydroxide or alkali-earth hydroxide counter-reactants can lead to the caking of milled powder and in turn repress the reaction kinetics to impede the completion of the reaction during milling, if the metal-hydroxide reaction-product decomposes into a metal oxide during milling to release water^83^.

Alkali-metal carbonate such as Na_2_CO_3_ can also work as a counter-reactant for metal-chloride raw materials to form water-soluble NaCl by-product. Na_2_CO_3_ has advantages over NaOH and Ca(OH)_2_ counter-reactants in the following aspects; (i) Na_2_CO_3_ is easier to handle than NaOH because it is less hygroscopic and corrosive than NaOH, (ii) Na_2_CO_3_ is softer than Ca(OH)_2_ thus easier to form a reactant nanocomposite during milling whereby reducing the milling time to complete the reaction, (iii) reaction products are metal carbonates which have moderate decomposition temperatures.

Na_2_CO_3_ was used for the first time in 1999 as a counter-reactant for metal-chloride raw materials to produce metal-oxide nanoparticles^71^. Since then, the use of alkali-metal carbonate counter-reactants has been successfully applied to produce a number of metal-oxide nanoparticles. For example, Fe_2_O_3_ nanoparticles were produced by mechanochemical processing via the reaction Fe_2_(SO_4_)_3_ + 3Na_2_CO_3_ → Fe_2_(CO_3_)_3_ + 3Na_2_SO_4_ and subsequent thermal decomposition^84^. The mixture of starting reactants Fe_2_(SO_4_)_3_, Na_2_CO_3_ and NaCl in a molar ratio of 1:3:4.3, was milled with a shaker-type mill. The NaCl diluent phase was added to decrease the volume fraction of Fe_2_O_3_ in the heat-treated powder down to 10%. The reaction was completed after milling for 4 h. After heat treatment at 400 °C for 1 hour and subsequent washing with water, Fe_2_O_3_ nanoparticles of ~6 nm in diameter was obtained. The particle-size distribution measured using electron micrographs was significantly narrow, with a standard deviation of only 3.3 nm. The result is in contrast with the use of FeCl_3_ and Ca(OH)_2_ where a longer milling time and the resulting Fe_2_O_3_ nanoparticles were polycrystalline and larger^77^.

The use of alkali-metal carbonate counter-reactants and an inert diluent has met success in producing other single-crystalline metal-oxide nanoparticles with a very small degree of agglomeration and narrow size distribution, including ZnO (~30 nm)^71^, SnO_2_ (5–30 nm)^71^, NiO (10 nm)^85^ and CdO (31 nm)^86^.

Most of the solid-state displacement reactions used in the mechanochemical synthesis of metal-oxide nanoparticles are associated with acid-base reactions. However, redox reactions can be also utilised (Table 1). For example, an attempt was made to mechanochemically react sodium dichromate and sulphur in a ball mill via the displacement reaction Na_2_Cr_2_O_7_ + S → Cr_2_O_3_ + Na_2_SO_4_ (dG = −521 kJ)^69^. Here, the starting material was not a metal salt but a tertiary metal oxide. The counter-reactant, S, selectively reduced Cr^6+^ into Cr^3+^ while being oxidised to S^6+^ to form a water-soluble sulphate by-product. After milling a stoichiometric mixture of the starting powders in a shaker mill for 10 min, an abrupt increase in the temperature of the milling vessel was observed^69^. In order to prevent the combustion event from occurring during milling, an inert diluent phase was added to the starting powder mixture. During the milling of a powder mixture Na_2_Cr_2_O_7_ + S + 7.8NaCl, the reaction progressed in a steady manner for 6 h and amorphous Cr_2_O_3_ particles with sizes around 4 nm were obtained^69^.

**Table 1:** Metal-oxide nanoparticles produced via mechanochemically induced redox reactions.

Material	Average size (nm)	Chemical reactions	Ref.
Cr_2_O_3_	4 nm	Na_2_Cr_2_O_7_ + S + 7.8NaCl → Cr_2_O_3_ + Na_2_SO_4_ + 7.8NaCl	^69^
MnO_2_	15–20 nm	2KMnO_4_ + MnCl_2_ → 3MnO_2_ + 2KCl + O_2_	^96^
Mn_2_O_3_	30 nm	2KMnO_4_ + 2NH_4_Cl → Mn_2_O_3_ + 2KCl + 4H_2_O + N_2_ + 0.5O_2_	^105^
CuMn_x_O_y_	50 nm	CuCl + xNaOH + yKMnO_4_ → CuMn_y_O_z_ + xNaCl + yKCl + 0.5xH_2_O	^106^

### Doped binary metal oxides

Many metal oxides are semiconductors and their optical and electrical properties can be tailored by impurity doping. Mechanochemical processing allows impurity doping of metal-oxide nanoparticles by adding a source of dopants in the starting powder mixture.

For example, ZnO nanoparticles doped with Mn or Co were synthesised by the mechanochemical activation of the reaction (1-x)ZnCl_2_ + xMCl_2_ + Na_2_CO_3_ + 4NaCl → Zn_1-x_M_x_CO_3_ + 6NaCl and subsequent heat treatment to decompose the resulting carbonate precursors into oxides, where M is the dopant element^87^. The concentration of dopants can be tailored by adjusting the molar fraction of the reactant materials in the starting powder mixture. It was found that the doping level can be continuously modified below 4 at% for Co-doping and 2 at% for Mn-doping^88^. However, the actual doping level measured in the nanoparticles was different from the nominal doping level in the starting powder mixture. The doping of Mn and Co reduced the photocatalytic activities of ZnO nanoparticles^87,88^. Other doped metal-oxide nanoparticles have been produced in a similar manner, as listed in Table 2. Since mechanochemical processing can lead to a state of high enthalpy, one can expect that the maximum doping level that mechanochemical processing can achieve may be higher than the other doping methods such as sol–gel techniques. However, no systematic investigation on the doping limit in mechanochemical processing has been reported to date.

**Table 2:** Doped metal-oxide nanoparticles produced using the mechanochemical activation of solid-state displacement reactions.

Material	Average size (nm)	Chemical reactions	Ref.
(Y_2_O_3_)_0.03_(ZrO_2_)_0.97_	15 nm	0.06YCl_3_ + 0.97ZrCl_4_ + 4.06LiOH → (Y_2_O_3_)_0.03_(ZrO_2_)_0.97_ + 4.06LiCl + 2.03H_2_O	^81^
(MgO)_0.1_(ZrO_2_)_0.9_	10 nm	0.1MgCl_2_ + 0.9ZrCl_4_ + 1.9Ca(OH)_2_ → (MgO)_0.1_(ZrO_2_)_0.9_ + 1.9CaCl_2_ + 1.9H_2_O	^107^
Co-doped ZnO	~30 nm	(1-x)ZnCl_2_ + xCoCl_2_ + Na_2_CO_3_ + 4NaCl → Zn_1-x_Co_x_O + 6NaCl + CO_2_	^87,88^
Mn-doped ZnO	~30 nm	(1-x)ZnCl_2_ + xMnCl_2_ + Na_2_CO_3_ + 4NaCl → Zn_1-x_Mn_x_O + 6NaCl + CO_2_	^87^
Mn-doped ZnO	~30 nm	(1-x)ZnCl_2_ + xMn(CH_3_COO)_2_ + Na_2_CO_3_ + 4NaCl → Zn_1-x_Mn_x_O + 6NaCl + xCH_3_COOH + CO_2_	^88^
Mn-doped SnO_2_	~30 nm	(1-x)SnCl_2_ + xMnCl_2_ + Na_2_CO_3_ + yNaCl → Sn_1-x_Mn_x_O + (2 + y)NaCl + CO_2_	^108^
Ag-doped ZnO	15–30 nm	(1-x) ZnCl_2_ + (1-x) Na_2_CO_3_ + xAg_2_O + 6NaCl → Zn_1-x_Ag_x_O + CO_2_ + 8NaCl	^109^
Al-doped ZnO	75–114 nm	(1-x)ZnCl_2_ + Na_2_CO_3_ + 8.6NaCl+xAl(NO_3_)_3_·9H_2_O → Zn_1-x_Al_x_O + 10.6NaCl + CO_2_	^110^

The list excludes the mechanochemical synthesis of doped metal oxides with non-nanostructures.

### Tertiary and other complex (high-entropy) metal oxides

Mechanochemical processing can be utilised to produce complex metal oxides such as tertiary oxides. The elemental composition can be controlled simply by the molar ratio of the reactant materials in the starting powder mixture. For example, Gagrani et al. produced Ca_2_Mn_3_O_8_, CaMn_2_O_4_ and CaMnO_3_ ultrafine particles by mechanochemical processing^89^. Ca_2_Mn_3_O_8_ nanoparticles were synthesised via ball milling of 2CaCl_2_ + 3MnCl_2_ + 5K_2_CO_3_ + 29KCl → Ca_2_Mn_3_(CO_3_)_5_ + 39KCl for 4 h and subsequent thermal decomposition of Ca_2_Mn_3_(CO_3_)_5_ into Ca_2_Mn_3_O_8_. The as-milled powder consisted of Ca_2_Mn_3_(CO_3_)_5_ nanoparticles of 10–20 nm in diameter. After heat treatment of the as-milled powder at 600 °C for 1 h, Ca_2_Mn_3_O_8_ nanoparticles had diameters in the range of 30–100 nm. CaMn_2_O_4_ and CaMnO_3_ nanoparticles were obtained in a similar manner, but using K_2_SO_4_ by-product phase instead of KCl^90^. This is because K_2_SO_4_ has a higher melting point (1069 °C) than KCl (770 °C) and the thermal decomposition of CaMn_2_(CO_3_)_3_ and CaMn(CO_3_)_2_ required heat treatment at higher than 770 °C. The mechanochemical reactions used were CaSO_4_ + 2MnSO_4_ + 3K_2_CO_3_ + 11K_2_SO_4_ → CaMn_2_(CO_3_)_3_ + 14K_2_SO_4_, and CaSO_4_ + MnSO_4_ + 2K_2_CO_3_ + 7K_2_SO_4_ → CaMn_2_(CO_3_)_3_ + 9K_2_SO_4_. In both cases, the amounts of extra K_2_SO_4_ added to the starting powder were such that the carbonates in the product phase had a volume fraction of 0.1. After milling for 4 h and subsequent heat treatment at 800 °C for 3 h, and 950 °C for 1 h, CaMnO_3_ (50–200 nm) and CaMn_2_O_4_ (200 nm–2 μm) were obtained, respectively.

Other tertiary metal-oxide ultrafine powders produced using the mechanochemical activation of solid-state displacement reactions are listed in Table 3.

**Table 3:** Tertiary metal-oxide nanoparticles produced using the mechanochemical activation of solid-state displacement reactions.

Material	Average size (nm)	Chemical reactions	Ref.
BaFe_12_O_19_	20–100 nm	1.2BaCl_2_ + 12FeCl_3_ + 38.4NaOH → BaFe_12_O_19_ + 38NaCl + 19H_2_O	^95^
ZnWO_4_	20–50 nm	H_2_WO_4_ + ZnCl_2_ + Na_2_CO_3_ + 4NaCl → ZnWO_4_ + 6NaCl + H_2_O + CO_2_	^111^
Ca_2_Mn_3_O_8_	10–20 nm	2CaCl_2_ + 3MnCl_2_ + 5K_2_CO_3_ + 29KCl → Ca_2_Mn_3_(CO_3_)_5_ + 39KCl; Ca_2_Mn_3_(CO_3_)_5_ + 1.5O_2_ → Ca_2_Mn_3_O_8_ + 5CO_2_	^89^
CaMn_2_O_4_	200nm-2μm	CaSO_4_ + 2MnSO_4_ + 3K_2_CO_3_ + 11K_2_SO_4_ → CaMn_2_(CO_3_)_3_ + 14K_2_SO_4_;CaMn_2_(CO_3_)_3_ + 0.5O_2_ → CaMn_2_O_4_ + 3CO_2_	^90^
CaMnO_3_	50–200 nm	CaSO_4_ + MnSO_4_ + 2K_2_CO_3_ + 7K_2_SO_4_ → CaMn(CO_3_)_2_ + 9K_2_SO_4_; CaMn(CO_3_)_2_ + 0.5O_2_ → CaMnO_3_ + 2CO_2_	^90^
CoFe_2_O_4_	5–100 nm	CoCl_2_ + 2FeCl_3_ + 8NaOH → CoFe_2_O_4_ + 8NaCl	^112^
LaCoO_3_	<100 nm	LaCl_3_ + CoCl_2_ + 5NaOH + xNaCl → La(OH)_3_ + Co(OH)_2_ + (5 + x)NaCl: La(OH)_3_ + Co(OH)_2_ + (5 + x)NaCl + 0.5O_2_ → LaCoO_3_ + (5 + x)NaCl + 2.5H_2_O	^113^
Ni_0.5_Zn_0.5_Fe_2_O_4_	30 nm	0.5NiCl_2_ + 0.5ZnCl_2_ + 2FeCl_3_ + 8NaOH → Ni_0.5_Zn_0.5_Fe_2_O_4_ + 8NaCl + 4H_2_O	^112^
La_0.7_Ca_0.3_MnO_3_	24 nm	0.7LaCl_3_ + 0.3CaCl_2_ + MnCl_2_ + 2.35Na_2_CO_3_ + 0.325O_2_ → La_0.7_Ca_0.3_MnO_3_ + 4.7NaCl + 2.35CO_2_	^114^

Nie et al.^91^ produced complex metal-oxide nanoparticles of 50–250 nm using mechanochemical processing. Metal acetate precursors and aluminium isopropoxide were milled together and subsequently heat-treated at 600 or 700 °C to form the so-called high-entropy metal-oxide nanoparticles consisting of more than five metal elements. It was found that the high-entropy nature imparts remarkable stability on particles, whose crystal structure and porosity can tolerate boiling water and high-temperature moisture.

Another approach to producing tertiary and high-enthalpy metal oxides by mechanochemical processing is mechanical alloying of binary metal oxides, metal carbonates or metal hydroxides. However, the approach does not utilise solid-state displacement reactions and thus is outside the scope of this review article.

### Size control

**Fig. 3:**
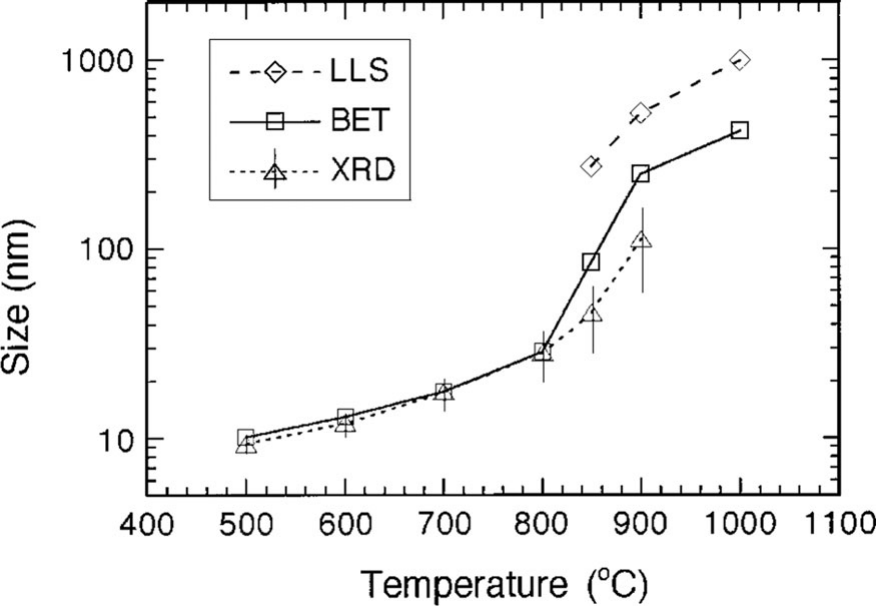
Particle sizes of mechanochemically synthesised CeO_2_ nanoparticles as a function of post-milling heat treatment temperature^70^. (Δ) Crystallite size estimated from the full-width at half maximum of X-ray diffraction (XRD) peaks, (⎕) particle size estimated from the Brunauer-–Emmett–Teller (BET) specific surface area, and (◊) particle size measured using a laser-light scattering (LLS) method. Reproduced with permission from John Wiley and Sons.

The control of particle size by varying milling-ball sizes and powder-to-matrix volume ratio has been described earlier in this review. The choice of reaction paths and reactants also influences the size of nanoparticles but the result is not predictable. Extending milling time beyond the reaction completion is another way to increase particle sizes through welding and aggregating the particles within a solid matrix. However, prolonged milling time will increase the defects in nanoparticles and, in an extreme case, transform the nanoparticles into amorphous. A more sensible option to increase particle size while retaining the low degree of agglomeration is to heat treat the as-milled powder. The solid matrix phase in the as-milled powder separates nanoparticles as long as the temperature is lower than the melting point of the matrix material. For example, the average size of CeO_2_ nanoparticles was tailored between 10 nm and 50 nm, by controlling post-milling heat-treatment temperature below the melting point of NaCl reaction by-product (Fig. 3)^70^. The proximity between particle sizes estimated using X-ray diffraction (XRD, crystallite size), Brunauer-Emmett-Teller (BET) specific surface area, and laser-light scattering (LLS, hydrodynamic diameter) has been demonstrated to be a good indicator to confirm that the low degree of agglomerations was retained during the size control^70^.

The mechanisms of particle-size control in solid matrices have not been elucidated. Theoretically, the solid barrier between nanoparticles should prevent nanoparticles from moving around to come into contact with each other, thus no particle growth should be expected. However, it was observed that a particle-growth behaviour mimics the Ostwald ripening during post-milling heat treatment^70^. It is speculated that the grain growth of the crystalline solid matrix plays a critical role in the size increase of nanoparticles during heat treatment. The increase in the size and crystallinity of the matrix’s crystal grains may coagulate nanoparticles, in a manner similar to ice crystals expelling gas molecules from their crystal lattice and gathering the gas molecules to certain locations when water freezes. However, direct evidence of this effect on nanoparticle growth in a solid matrix has not been documented.

### Shape control

The shape of nanoparticles is important to some of their properties. For example, the exposure of selected crystal facets increases or decreases the catalytic activities of nanoparticles, because the surface energy depends on the crystal facets^92^. It was demonstrated that the shape of mechanochemically produced metal-oxide nanoparticles can be controlled to a certain degree. The operation involves heat treatment of as-milled powders at elevated temperatures while nanoparticles are still embedded in a by-product or diluent phase.

**Fig. 4:**
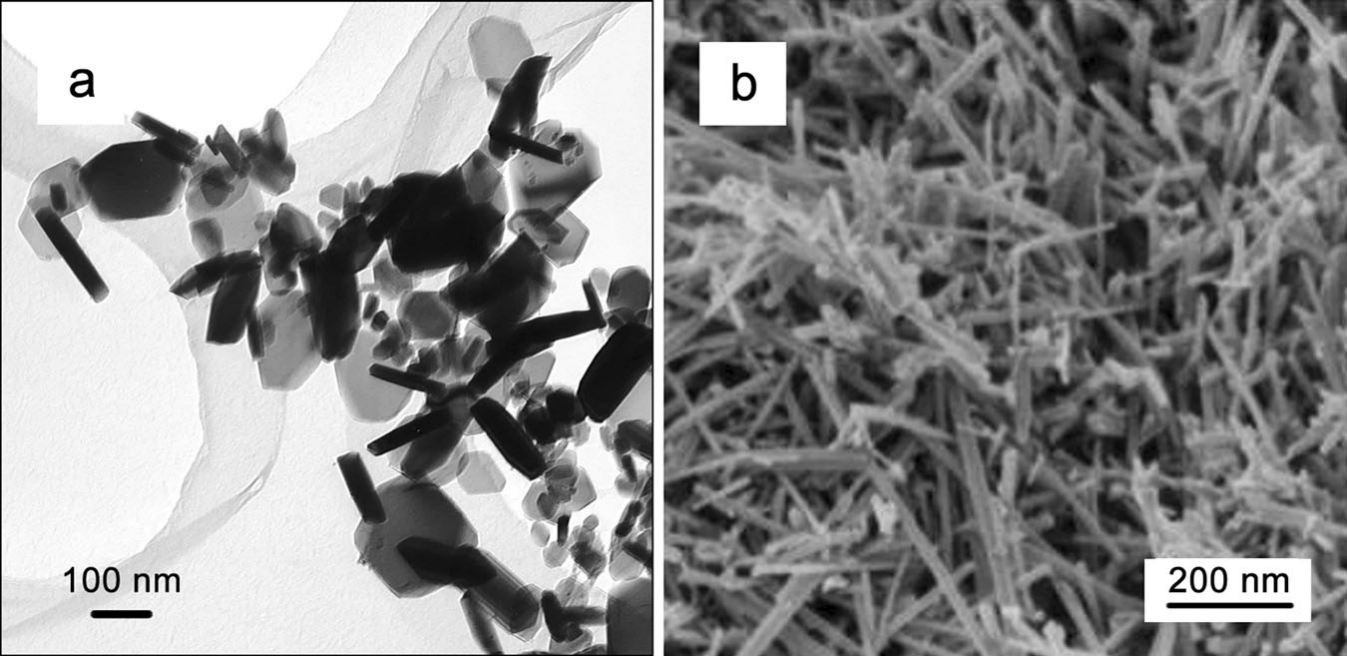
Examples of morphology-controlled nanoparticles produced by mechanochemical processing. **a** Transmission electron micrograph of α-Fe_2_O_3_ plate-shaped nanoparticles^93^. **b** Scanning electron micrograph of MnO_2_ nanorods^96^. Reproduced with permission from Elsevier.

Well-disperse α-Fe_2_O_3_ (haematite) nanoparticles with a plate-shape were produced from the γ-Fe_2_O_3_ (~6 nm) that were mechanochemically synthesised via the reaction Fe_2_(SO_4_)_3_ + 3Na_2_CO_3_ + 4.3NaCl → γ-Fe_2_O_3_ + 3Na_2_SO_4_ + 4.3NaCl + 3CO_2_^93^. Prior to removing Na_2_SO_4_ and NaCl, the γ-Fe_2_O_3_ nanopowder was heat-treated at 700^o^C in the air for 1 h. The resulting α-Fe_2_O_3_ consisted of only hexagonal platelets having diameters of 50–200 nm and thicknesses of about 20–40 nm, with a low degree of agglomeration (Fig. 4a). The heat treatment temperature of 700 °C was above the eutectic melting temperature of Na_2_SO_4_ and NaCl mixture (~620 °C) and the molten salts may have acted as a flux to form the platey shapes of haematite^94^. Nevertheless, this new synthesis route of Fe_2_O_3_ platelets can produce much smaller particles than conventional molten-salt techniques and did not require the addition of any mineralizers that are normally necessary to form ceramic nanoparticles with unique shapes^94^.

BaFe_12_O_19_ magnetic nano-platelets were produced in a similar manner:^95^ Ball milling of 1.2BaCl_2_ + 12FeCl_3_ + 38.4NaOH + 200 wt% NaCl induced a chemical reaction to form metal hydroxides and NaCl. The as-milled powder was heat-treated at ~800 °C, at the melting point of NaCl, in the air for 1 h, resulting in BaFe_12_O_19_ platelet nanoparticles with diameters of 20–100 nm and thicknesses of 10–20 nm.

The shape control of MnO_2_ nanoparticles was achieved at a temperature much lower than the melting point of a slat matrix phase^96^. In the study, MnO_2_ was produced via the mechanochemical reaction 2KMnO_4_ + MnCl_2_ → 3MnO_2_ + 2KCl + O_2_ with the addition of KCl diluent, and the as-milled powder was heated at 350 °C for 1 h to obtain single-crystalline nanorods of 15–20 nm in diameter and 100–400 nm in length (Fig. 4b). This temperature is significantly lower than the melting temperature of NaCl (801 °C). As such, the morphology-transformation cannot be explained using existing knowledge such as the molten-salt crystal-growth mechanism^97^.

Interestingly, similar processes do not always lead to shaped nanoparticles. For example, CeO_2_ nanoparticles of ~10 nm in diameter were produced in a NaCl matrix via a mechanochemical reaction CeCl_3_ + 3NaOH + 12NaCl → Ce(OH)_3_ + 15NaCl and the effect of heat treatment temperature on the particle size of CeO_2_ was studied^70^. The heat treatment produced only near-spherical particles, regardless of the temperature being below or above the melting point of NaCl. The mechanisms of the particle-shape control in post-milling heat treatment have not been fully understood.

### Soft mechanochemistry

Soft mechanochemical reactions involve highly reactive raw materials, often represented by hydrate or hydroxide materials^98^. A comprehensive review of this synthesis approach is given by Avvakumov et al.^99^ and the theoretical explanation of the process was made from the viewpoint of hydrothermal reactions by Boldyrev^100^. Some consider the use of hydroxide counter-reactants such as NaOH as an example of soft-mechanochemical processing. Hydrate or hydroxide materials tend to be softer than anhydrous or oxide forms of materials, which benefits the mechanochemical induction of chemical reactions and, in turn, assists in the production of nanoscale materials^101^. It is believed that soft mechanochemistry plays an important role in biological processes in nature^102^.

A variety of soft-mechanochemical displacement reactions were used to produce many binary, tertiary and doped metal oxides (Table 4). Generally, the use of hydrous metal salts enables shorter milling times (< 1 h) to complete the reaction than anhydrous raw materials. In addition, hydrate materials tend to be less expensive than anhydrous materials and more readily available. Hence soft-mechanochemical processing may be more beneficial than conventional mechanochemical processing in terms of reduced environmental footprint^24^.

**Table 4:** Examples of metal-oxide nanoparticles produced by soft-mechanochemical processing (not a comprehensive list).

Material	Average size (nm)	Chemical reactions	Ref.
SiO_2_	34 nm	Na_2_SiO_3_·9H_2_O + 2NH_4_Cl → _2NaCl + 2NH_3_ + 9H_2_O + SiO_2_	^115^
CeO_2_	2.5 nm	(NH_4_)_2_Ce(NO_3_)_6_ + 6NaOH → 6NaNO_3_ + 2NH_3_ + 4H_2_O + CeO_2_	^115^
CeO_2_	40–70 nm	2CeCl_3_·6H_2_O + 3Na_2_CO_3_·10H_2_O → 2CeO_2_ + 6NaCl + 2CO_2_ + 42H_2_O	^116^
SnO_2_	1.0 nm	SnCl_4_·5H_2_O + 4NaOH → 4NaCl + 7H_2_O + SnO_2_	^115^
γ-Fe_2_O_3_	~10 nm	2FeCl_3_·6H_2_O + 6NaOH → γ-Fe_2_O_3_ + 6NaCl + 15H_2_O	^117^
CuO	Aggregates	CuCl_2_·2H_2_O + NaOH +xNaCl →CuO + (x + 2)NaCl + 3H_2_O	^118^
Co_3_O_4_	13 nm	2Co(NO_3_)_2_·6H_2_O + 5NH_4_HCO_3_ → Co_2_(OH)_2_CO_3_ + 4NH_4_NO_3_ + NH_3_ + 4CO_2_ + 14H_2_O; Co_2_(OH)_2_CO_3_ + O_2_ → Ce_3_O_4_ + CO_2_ + H_2_O	^119^
In_2_O_3_	27 nm	2InCl_3_·4H_2_O + 6NaOH → In_2_O_3_ + 6NaCl + 8H_2_O	^120^
CoFe_2_O_4_	10 nm	CoCl_2_·6H_2_O + 2FeCl_3_·6H_2_O + 8KOH + xNaCl → CoFe_2_O_4_ + 8KCl+ xNaCl + 22H_2_O	^121^
Se-doped ZnO	10 nm	(1-x) [Zn(CH_3_COO)_2_·2H_2_O + (COOH)_2_·2H_2_O] + xSe + yO_2_ → Zn_1-x_Se_x_O + (2–2x)CH_3_COOH + (4–2.5x) H_2_O + (2–2x)CO_2_	^122^
B-doped ZnO	15 nm	(1-x) [Zn(CH_3_COO)_2_·2H_2_O + (COOH)_2_·2H_2_O] + xH_3_BO_3_ + yO_2_ → Zn_1-x_B_x_O + (2–2x)CH_3_COOH + (4–2.5x) H_2_O + (2–2x)CO_2_	^123^

## Conclusions and outlook

In the last three decades, the development of mechanochemical processing to synthesise metal-oxide nanoparticles have made significant progress. The versatile nature of mechanochemical processing was demonstrated for its ability to produce a variety of metal-oxide nanoparticles including doped oxides and complex oxide, as well as the ability to produce single-crystalline nanoparticles with little agglomeration in a manner the particle size and shapes are controlled. In particular, mechanochemistry offers positive aspects of green chemistry, which has started to attract wide recognition in recent years. Although mechanochemistry has become a research field of its own, mechanochemical processing of metal-oxide nanoparticles has not yet reached its maturity. This article reviewed the progress of the research field of the mechanochemical synthesis of metal-oxide nanoparticles, by addressing the critical influencing factors such as the choice of reaction pathways and selection of reactants, whereby providing new insights into existing research gaps that could serve as a guideline for future research necessary to take the technology further.

For example, the effects of process parameters require further investigation. The combination of ball-to-powder ratio and milling time can influence the frequency of reactant-ball collision events and thus affect the time to complete the reaction. However, the influence of the combination on the nanoparticle end products has not been documented. Also, the influence of mill types on the kinetics of mechanochemical synthesis of metal-oxide nanoparticles has not been understood. Shaker mills mostly give impact-type force to the reactant powders whilst shear force is dominated in planetary mills and attrition mills. The deformation, fracture and welding of reactant crystals may occur in different ways under different types of forces, as demonstrated in the mechanochemical co-crystallisation of organic compounds^16^. The study on the topic will shed more light on the mechanisms of mechanochemical synthesis of metal-oxide nanoparticles.

In addition, the mechanisms of the particle-shape control in post-milling heat treatment are not fully understood. It is speculated that the salt matrix may influence the surface energy of certain crystal facets of metal oxides to assist the shaping of particles^103^. The ability to shape nanoparticles may depend on not only temperature but also the interaction between the ions in the salt matrix and metal-oxide surface and also impurity migration into the crystals. Further study on these points is required to elucidate the mechanisms of nanoparticle shaping within a salt matrix. This will expand the ability of mechanochemical processing to produce nanoparticles with a variety of shapes including belt, cube, disk, hollow-shell and Janus structures.

The assumption that NaCl behaves as an inert diluent phase in many mechanochemical reactions, may require re-examination. The study of Shu et al.^73^ indicated that metal-chloride stating reactants can mechanochemically form a solid solution. It should be investigated whether the reaction pathways may involve the formation of solid solutions between a diluent phase and metal-halide raw materials before the solid solution reacts with the counter-reactant to form metal-oxide nanoparticles. Most of the past studies on the mechanochemical formation of solid solutions are limited to metal alloys (with metallic bonds) or metal oxides (with covalent bonds being dominant). If the mechanochemical formation of solid solutions of ionic crystals can be widely demonstrated using many types of ionic crystals, that may open up a new avenue for mechanochemistry to expand its applications in the synthesis of new materials.

Although soft-mechanochemical processing has some advantages over non-soft-mechanochemical processing, the methods to control size, shape and degree of agglomeration using soft mechanochemical processing are under-developed compared to non-soft-mechanochemical processing. The approaches taken for non-soft-mechanochemical processing described in this review may be useful in this aspect. In addition, many studies of soft-mechanochemical synthesis require re-visiting by taking into account the following precautions: (i) many hydrous metal salts are highly hygroscopic and thus, if the milling chamber is not sealed, the effects of atmospheric humidity on the reaction kinetics is difficult to determine; (ii) hydrous metal salts are mostly soluble so that the occurrence of mechanochemical process should be confirmed during milling or before washing the as-milled powder; (iii) the milling of hydrous metal salts tends to become amorphous so that the completion of the reaction before heat treatment or by-product removal is difficult to assess using XRD; (iv) hydrous metal salts may release water during milling^83^ and cake the milled powder within the milling container, reducing the milling efficiency or altering the reaction path (e.g. liquid-assisted grinding^104^); (v) the method normally results in the production of precursors that require thermal decomposition to form metal oxides and the heat treatment may agglomerate nanoparticles in the absence of an appropriate quantity of an inert matrix phase; (iv) the involvement of water and corrosive raw materials may introduce impurities arising from the corrosion of milling container and balls.

Furthermore, the analysis of environmental impacts in mechanochemical processing should be conducted to confirm its claim for green chemistry. The analysis may be difficult, as many different conditions are used in mechanochemical processing and the number of synthesis conditions is rapidly increasing. Nonetheless, the determination of reaction path, mill type and energy input should enable one to perform reasonable life-cycle analysis. This would assist in manifesting the potential of mechanochemistry in the sustainable green production of metal-oxide nanoparticles.
